# Telomere Length and Risk of Incident Fracture and Arthroplasty: Findings From UK Biobank

**DOI:** 10.1002/jbmr.4664

**Published:** 2022-09-13

**Authors:** Elizabeth M. Curtis, Veryan Codd, Christopher Nelson, Stefania D'Angelo, Qingning Wang, Elias Allara, Stephen Kaptoge, Paul M. Matthews, Jonathan H. Tobias, John Danesh, Cyrus Cooper, Nilesh J. Samani, Nicholas C. Harvey

**Affiliations:** ^1^ MRC Lifecourse Epidemiology Centre University of Southampton Southampton UK; ^2^ Department of Cardiovascular Sciences University of Leicester Leicester UK; ^3^ NIHR Leicester Biomedical Research Centre Glenfield Hospital Leicester UK; ^4^ British Heart Foundation Cardiovascular Epidemiology Unit, Department of Public Health and Primary Care University of Cambridge Cambridge UK; ^5^ National Institute for Health Research Blood and Transplant Research Unit in Donor Health and Genomics University of Cambridge Cambridge UK; ^6^ British Heart Foundation Centre of Research Excellence University of Cambridge Cambridge UK; ^7^ Department of Brain Sciences and UK Dementia Research Institute Centre Imperial College London London UK; ^8^ Musculoskeletal Research Unit University of Bristol Bristol UK; ^9^ Medical Research Council Integrative Epidemiology Unit University of Bristol Bristol UK; ^10^ Health Data Research UK Cambridge Wellcome Genome Campus and University of Cambridge Cambridge UK; ^11^ Department of Human Genetics Wellcome Sanger Institute Hinxton UK; ^12^ National Institute for Health and Care Research (NIHR) Southampton Biomedical Research Centre University of Southampton and University Hospital Southampton NHS Foundation Trust Southampton UK; ^13^ National Institute for Health and Care Research (NIHR) Oxford Biomedical Research Centre University of Oxford Oxford UK

**Keywords:** OSTEOPOROSIS, OSTEOARTHRITIS, EPIDEMIOLOGY, LEUCOCYTE TELOMERE LENGTH, AGING

## Abstract

We investigated independent associations between telomere length and risk of fracture and arthroplasty in UK Biobank participants. Leukocyte telomere length (LTL) was measured in baseline samples using a validated polymerase chain reaction (PCR) method. We used, in men and women separately, Cox proportional hazards models to calculate the hazard ratio (HR) for incident fracture (any, osteoporotic) or arthroplasty (hip or knee) over 1,186,410 person‐years of follow‐up. Covariates included age, white cell count, ethnicity, smoking, alcohol, physical activity, and menopause (women). In further analyses we adjusted for either estimated bone mineral density (eBMD) from heel quantitative ultrasound, handgrip strength, gait speed, total fat mass (bioimpedance), or blood biomarkers, all measured at baseline (2006–2010). We studied 59,500 women and 51,895 men, mean ± standard deviation (SD) age 56.4 ± 8.0 and 57.0 ± 8.3 years, respectively. During follow‐up there were 5619 fractures; 5285 hip and 4261 knee arthroplasties. In confounder‐adjusted models, longer LTL was associated with reduced risk of incident knee arthroplasty in both men (HR/SD 0.93; 95% confidence interval [CI], 0.88–0.97) and women (0.92; 95% CI, 0.88–0.96), and hip arthroplasty in men (0.91; 95% CI, 0.87–0.95), but not women (0.98; 95% CI, 0.94–1.01). Longer LTL was weakly associated with reduced risk of any incident fracture in women (HR/SD 0.96; 95% CI, 0.93–1.00) with less evidence in men (0.98; 95% CI, 0.93–1.02). Associations with incident outcomes were not materially altered by adjustment for heel eBMD, grip strength, gait speed, fat mass, or blood biomarker measures. In this, the largest study to date, longer LTL was associated with lower risk of incident knee or hip arthroplasty, but only weakly associated with lower risk of fracture. The relative risks were low at a population level, but our findings suggest that common factors acting on the myeloid and musculoskeletal systems might influence later life musculoskeletal outcomes. © 2022 The Authors. *Journal of Bone and Mineral Research* published by Wiley Periodicals LLC on behalf of American Society for Bone and Mineral Research (ASBMR).

## Introduction

Telomeres are DNA‐protein structures found at the ends of chromosomes that protect the genome from damage, and are made up of a large number of tandem repeats of a simple DNA sequence (in humans TTAGGG). They have been proposed as potential markers of biological aging,^(^ ^1^, ^2^ ^)^ because they have been shown to shorten progressively over time in most somatic tissues.^(^ ^3^ ^)^ Measured in peripheral blood leucocytes, shorter telomeres are correlated with male sex, older age, and other known risk factors for noncommunicable diseases.^(^ ^4^, ^5^ ^)^ They have also been shown to be generally associated with greater risk of cardiovascular disease,^(^ ^6^ ^)^ type 2 diabetes,^(^ ^7^ ^)^ and nonvascular, non‐cancer causes of mortality.^(^ ^8^ ^)^

Common chronic noncommunicable musculoskeletal disorders, such as osteoporosis, osteoarthritis, and sarcopenia, become more common with age and are associated with considerable morbidity and mortality (particularly following hip fracture).^(^ ^9^, ^10^ ^)^ Telomere length has been shown to be associated with bone mineral density (BMD) and osteoporosis,^(^ ^11^, ^12^ ^)^ the aging of articular cartilage,^(^ ^13^ ^)^ and grip strength^(^ ^14^ ^)^ in some studies, with others showing no association with BMD or physical performance.^(^ ^15^, ^16^ ^)^ The need for larger studies investigating associations between telomere length and musculoskeletal health has been recognized, particularly for understanding the underlying mechanisms.^(^ ^17^ ^)^ With its unparalleled sample size, and intensive phenotyping of participants' musculoskeletal and broader health measures, UK Biobank (UKB) permits robust investigation of such associations and the potential underlying mechanisms. The aim of this study was therefore to investigate associations between telomere length for age and risk of incident fracture (the consequence of osteoporosis) and arthroplasty (the consequence of osteoarthritis), adjusting for a comprehensive range of confounding factors and evaluating the role of measures related to bone, body composition, and blood biochemical markers.

## Subjects and Methods

### Setting and recruitment

UKB is a population study incorporating over half a million participants recruited 2006–2010 from across the UK.^(^ ^18^ ^)^ Individuals aged 40–69 years were identified through National Health Service (NHS) registers and invited to participate. The baseline assessment, undertaken at regional centers (2006–2010), included detailed review of demographics, lifestyle, medical history, a series of physical measures, and blood sampling. The protocol is publicly available.^(^ ^18^ ^)^ Individuals who were unable to consent or complete baseline assessment due to illness or discomfort were not recruited. Linkages with Hospital Episode Statistics (HES) and death registers enable longitudinal tracking of health outcomes for all participants.

### Ethics

This study is covered by the ethical approval for UK Biobank studies from the NHS National Research Ethics Service on 17th June 2011 (Ref 11/NW/0382) and extended 10th May 2016 (Ref 16/NW/0274).

### Exposure: Leukocyte telomere length

Leukocyte telomere length (LTL) was measured in the whole cohort at baseline using a validated polymerase chain reaction (PCR) method that expresses LTL as the ratio of telomere repeat copy number (T) relative to that of a single copy gene (S, Hgb) (T/S ratio).^(^ ^19^ ^)^ LTL measurements were adjusted for technical variation, log_e_ transformed, and *Z*‐standardized. Paired LTL measurements at two time‐points (mean interval, 5.5 years) in 1351 participants yielded a regression‐dilution ratio of ~0.68.^(^ ^19^ ^)^

### Other baseline measures

Age, sex, ethnicity, education, smoking, alcohol intake, and menopause status were recorded at baseline via self‐report (touchscreen questionnaire) and interview. Physical activity was recorded at baseline as the number of days per week a participant engages in vigorous physical activity for at least 10 minutes. Material deprivation was recorded as the Townsend index. Directly measured gait speed was not available in this cohort so we used questionnaire‐assessed usual gait speed, after establishing that it was predictive of fracture outcomes consistent with the direct measure obtained in a previous study.^(^ ^20^ ^)^ Body mass index (BMI) was calculated from height and weight recorded at baseline. Total body fat mass was measured using bioimpedance (Tanita Europe, Amsterdam, NL), the measure that has been shown to correlate well with dual‐energy X‐ray absorptiometry (DXA) total fat mass.^(^ ^21^, ^22^ ^)^ An estimate of bone mineral density (eBMD) at the heel was obtained using heel quantitative ultrasound scanning, using the Sahara Clinical Bone Sonometer (Hologic, Inc., Marlborough, MA, USA) according to a predefined standard operating procedure.^(^ ^23^ ^)^ This technique has been shown to generate a measure predictive of incident fracture of comparable, if somewhat lower, effect size to that from DXA.^(^ ^24^ ^)^ Grip strength was measured in both left and right hands using a Jamar J00105 hydraulic hand dynamometer (Lafayette Instrument, Lafayette, IN, USA) with the maximum value used for analysis.

We considered the following serum biochemistry measures (from bloods collected at the baseline visit) as potential biological mediators: C‐reactive protein (CRP), creatinine, vitamin D, calcium, insulin‐like growth factor‐1 (IGF1), sex hormone binding globulin (SHBG), testosterone, testosterone/SHBG, estradiol, phosphate, and cystatin C. Details of the biochemical methods are available on the UK biobank website (https://www.ukbiobank.ac.uk/media/oiudpjqa/bcm023_ukb_biomarker_panel_website_v1‐0‐aug‐2015‐edit‐2018.pdf).

### Outcomes: Incident fractures and arthroplasty

We ascertained incident fractures and arthroplasty (at the knee or hip) from linkage to HES using predefined International Classification of Diseases and Related Health Problems, 10th Revision (ICD‐10) categories (fractures) or UK Operating Procedure Codes (OPCS; for arthroplasty). The codes considered for either outcome type are listed in Table S1. Fracture sites considered included the skull, vertebra, rib, pelvis, clavicle, scapula, humerus, radius/ulna, carpus, femur/hip, patella, tibia/fibula, ankle, foot, or unspecified fractures. We grouped fracture outcomes as all or osteoporotic (clinical vertebral, ribs, pelvis, humerus, clavicle, scapula, sternum, hip, other femoral fractures, tibia, fibula, and distal forearm/wrist).^(^ ^25^ ^)^ Arthroplasty was examined separately at the knee and hip. We obtained information on mortality from the UK Office for National Statistics (ONS) death registry linkage.

### Statistical analysis

We described baseline characteristics by reporting mean ± standard deviation (SD) or median (interquartile range [IQR]) as appropriate for continuous variables, and number (percentages) for categorical variables. Differences between groups were tested with unpaired *t* tests, Mann‐Whitney *U* tests, or Pearson chi‐square tests, as appropriate. We undertook analyses separately in men and women, testing for interactions by sex.

We used Cox proportional hazards models to investigate associations between LTL and risk of incident fracture or arthroplasty, presenting the relationship as a hazard ratio (HR) per SD increase in LTL. The primary analyses were based on the subset of participants who had complete data for the exposure, outcomes, and covariates. The proportional hazards assumption was met for the exposure with all outcomes other than for the arthroplasty in men. Further diagnostic investigation stratifying associations by quarters of LTL, and by covariate status (for covariates where the assumption was also not met), suggested that these deviations would not alter the interpretation of the findings, and are presented in Tables S8 and S9. Time at risk was right censored at either the first incident event, death, or November 30, 2020 (end of observation period; same for all data sources). On the basis of prior literature and biological plausibility, we included the following covariates in our fully adjusted models (ie, considering them as true confounders; model 1): chronological age, age squared, white cell count, ethnicity, smoking, alcohol, physical activity, and menopause (women). Figure S2 documents the relationship between LTL and chronological age. The inclusion of both chronological age and age squared was found to provide better age‐adjustment than including the first‐order term alone. In further exploratory models we investigated whether addition of other measures associated with musculoskeletal health, and potentially on the causal pathway, altered the relationship between exposure and outcome, considering eBMD from heel quantitative ultrasound, grip strength, gait speed, total fat mass, or blood biomarkers. The final fully adjusted model (model 2) includes all of these factors. The blood biomarkers were selected for inclusion in these models on the basis of associations with LTL (Table S2). We investigated potential nonlinearity in the LTL‐outcome associations by stratifying the relationships by quarters of LTL. In a sensitivity analysis, we repeated the analyses using the maximum numbers available for each test; in a further sensitivity analysis, we used the regression‐dilution ratio to account for within‐person variability of LTL values over time (abbreviated “usual LTL”), as described.^(^ ^19^ ^)^

## Results

### Characteristics of the participants

The analysis dataset comprised 111,452 individuals, mean ± SD age 56.5 ± 8.1 years. A total of 59,500 were women (53.4%), with mean ± SD age 56.4 ± 8.0 years; there were thus 51,895 men, mean ± SD age 57.0 ± 8.3 years. Over 90% (94.1%) of men and 94.3% of women described themselves as being of white ethnicity; the median body mass index in men and women was 27.3 and 26.0 kg/m^2^, respectively. During 1,186,410 person‐years of follow‐up, there were 5619 fractures, 5285 hip arthroplasties, and 4261 knee arthroplasties. Table 1 summarizes the baseline characteristics of the cohort. Figure S1 documents the distribution of the exposure variable, both raw and standardized.

**Table 1:** Characteristics of Participants

Characteristic	Women (*n* = 59,500)	Men (*n* = 51,895)
Age at baseline (years), mean ± SD	56.4 (8.0)	57.0 (8.3)
BMI (kg/m^2^), median (IQR)	26.0 (23.4,29.6)	27.3 (25.0,30.1)
Ethnicity (white), *n* (%)	55282 (92.9)	48109 (92.7)
Smoking status, *n* (%)		
Never	35446 (59.6)	25723 (49.6)
Previous	19139 (32.2)	20126 (38.8)
Current	4915 (8.3)	6046 (11.7)
Alcohol consumption, *n* (%)		
Daily or almost daily	9777 (16.4)	13505 (26.0)
Three or four times a week	12163 (20.4)	13257 (25.6)
Once or twice a week	14981 (25.2)	13279 (25.6)
One to three times a month	7910 (13.3)	4730 (9.1)
Special occasions only	9059 (15.2)	3836 (7.4)
Never	5610 (9.4)	3288 (6.3)
Level of education, *n* (%)		
College/university	19843 (33.4)	18692 (36.0)
Other professional qualifications/HND/A levels	19462 (32.7)	18064 (34.8)
GCSE or less	19780 (33.2)	14698 (28.3)
Missing	415 (0.7)	441 (0.9)
Days/week vigorous physical activity, median (IQR)	1 (0,3)	2 (0,3)
Postmenopause, *n* (%)	36547 (61.4)	–
Gait speed at baseline, *n* (%)		
Slow	4420 (7.4)	3626 (7.0)
Normal	31566 (53.1)	27314 (52.6)
Fast	23514 (39.5)	20955 (40.4)
Maximum grip strength at baseline (kg), mean ± SD	24.5 (6.2)	40.7 (8.9)
Heel BMD at baseline (g/cm^2^), mean ± SD	0.52 (0.12)	0.58 (0.14)
Follow‐up time (person‐years)	631072.3	555338.4
Any incident fracture, *n* (%)	3495 (5.9)	2124 (4.1)
Any incident osteoporotic fracture, *n* (%)	2426 (4.1)	1333 (2.6)
Any incident major osteoporotic fracture, *n* (%)	1804 (3.0)	696 (1.3)
Incident arthroplasty at hip (primary or revision), *n* (%)	3113 (5.2)	2145 (4.1)
Incident arthroplasty at knee (primary or revision), *n* (%)	2232 (3.8)	1930 (3.7)
Incident arthroplasty at hip (primary only), *n* (%)	3006 (5.1)	2088 (4.0)
Incident arthroplasty at knee (primary only), *n* (%)	2192 (3.7)	1880 (3.6)
Death, *n* (%)	239 (0.4)	295 (0.6)

GCSE = General Certificate of Secondary Education; HND = Higher National Diploma.

### Associations between LTL and incident fractures

In the confounder‐adjusted model (model 1), there was weak evidence for an association between greater LTL and a lower risk of any incident fracture in women (HR/SD 0.96; 95% CI, 0.93–1.00), but less so in men (HR/SD 0.98; 95% CI, 0.93–1.02). A similar pattern was observed with the outcome of incident osteoporotic fractures, again with the association in men (0.98; 95% CI, 0.92–1.03) similar (but of lesser magnitude) to that in women (0.96; 95% CI, 0.92–1.00). Consistent with these findings, there was evidence of a sex interaction (any incident fracture, *p* = 0.03 and osteoporotic fracture, *p* = 0.10). These associations are summarized in Table 2, which also documents the further exploratory models incorporating adjustment for heel eBMD, grip strength, usual gait speed, total body fat mass, or blood biomarkers. Addition of these measures did not materially alter the association between LTL and either any incident fracture or incident osteoporotic fracture. Tables S3 and S6 document the similar findings in sensitivity analyses using the maximum number of individuals for each test with or without adjustment for regression dilution. Table S8 demonstrates that although the HR point estimates showed some modest differences according to quarter of LTL, the 95% CIs overlapped, such that there was no convincing evidence of nonlinear relationships.

**Table 2:** Associations between LTL and Incident Fractures

	All (*n* = 111,452)		Women (*n* = 59,500)		Men (*n* = 51,895)		*p* sex interaction
Variable	HR (95% CI)	*p*	HR (95% CI)	*p*	HR (95% CI)	*p*	
Any incident fracture							
Unadjusted	0.93 (0.91–0.95)	<0.001	0.89 (0.86–0.92)	<0.001	0.95 (0.91–0.99)	0.01	0.03
Model 1	0.99 (0.96–1.02)	0.42	0.96 (0.93–1.00)	0.04	0.98 (0.93–1.02)	0.29	
Model 1+ heel BMD	0.98 (0.95–1.01)	0.15	0.97 (0.93–1.00)	0.05	0.98 (0.93–1.02)	0.26	
Model 1+ grip strength	0.98 (0.95–1.00)	0.08	0.97 (0.93–1.00)	0.05	0.98 (0.93–1.02)	0.29	
Model 1+ gait speed	0.99 (0.97–1.02)	0.55	0.97 (0.93–1.00)	0.05	0.98 (0.94–1.02)	0.36	
Model 1+ total fat (bioimpedance)	0.99 (0.96–1.02)	0.42	0.96 (0.93–1.00)	0.04	0.98 (0.93–1.02)	0.27	
Model 1+ blood biomarkers	0.98 (0.95–1.00)	0.10	0.97 (0.93–1.00)	0.05	0.98 (0.94–1.03)	0.48	
Model 2	0.98 (0.95–1.00)	0.08	0.97 (0.94–1.01)	0.10	0.99 (0.94–1.03)	0.52	
Any incident osteoporotic fracture							
Unadjusted	0.91 (0.88–0.94)	<0.001	0.87 (0.84–0.91)	<0.001	0.92 (0.87–0.97)	0.003	0.10
Model 1	0.99 (0.96–1.02)	0.45	0.96 (0.92–1.00)	0.03	0.98 (0.92.1.03)	0.37	
Model 1+ heel BMD	0.98 (0.94–1.01)	0.15	0.96 (0.92–1.00)	0.05	0.97 (0.92–1.03)	0.34	
Model 1+ grip strength	0.97 (0.94–1.00)	0.09	0.96 (0.92–1.00)	0.04	0.98 (0.92–1.03)	0.38	
Model 1+ gait speed	0.99 (0.96–1.02)	0.55	0.96 (0.92–1.00)	0.04	0.98 (0.93–1.03)	0.45	
Model 1+ total fat (bioimpedance)	0.99 (0.96–1.02)	0.44	0.95 (0.92–0.99)	0.02	0.97 (0.92–1.03)	0.32	
Model 1+ blood biomarkers	0.97 (0.94–1.00)	0.09	0.96 (0.92–1.00)	0.04	0.98 (0.93–1.04)	0.56	
Model 2	0.97 (0.94–1.00)	0.07	0.96 (0.93–1.00)	0.08	0.98 (0.93–1.04)	0.57	

Model 1: adjusted for age, age quadratic, white cell count, ethnicity, smoking, alcohol, physical activity and menopause (women). Blood biomarkers included are: C‐reactive protein, calcium, alkaline phosphatase, urate, SHBG, creatinine, cystatin C, HbA1c. Model 2 = model 1+ heel BMD, grip strength, gait speed, total fat, blood biomarkers.

### Associations between LTL and incident arthroplasty

In confounder‐adjusted models (model 1), summarized in Table 3, longer LTL was associated with reduced risk of incident knee arthroplasty in both men (HR/SD 0.93; 95% CI, 0.88–0.97) and women (0.92; 95% CI, 0.88–0.96) and hip arthroplasty in men (0.91; 95% CI, 0.87–0.95) but not women (0.98; 95% CI, 0.94–1.01). Further adjustment for heel eBMD, grip strength, total fat mass, or blood biomarkers again did not materially change the associations. In sensitivity analyses, findings were similar when only primary arthroplasty was used as the outcome at either site (Table S4), and for analyses with the maximum sample size adjusted or unadjusted for the regression dilution ratio (Tables S4, S5, and S7). Again there was little evidence of convincing nonlinearity (Table S8).

**Table 3:** Associations Between LTL and Incident Arthroplasty

	All (*n* = 111,452)		Women (*n* = 59,500)		Men (*n* = 51,895)		*p* sex interaction
Variable	HR (95% CI)	*p*	HR (95% CI)	*p*	HR (95% CI)	*p*	
Incident hip arthroplasty							
Unadjusted	0.86 (0.84–0.88)	<0.001	0.88 (0.85–0.91)	<0.001	0.81 (0.78–0.85)	<0.001	0.006
Model 1	0.96 (0.93–0.99)	0.42	0.98 (0.94–1.01)	0.20	0.91 (0.87–0.95)	<0.001	
Model 1+ heel BMD	0.96 (0.94–0.99)	0.15	0.98 (0.94–1.01)	0.19	0.91 (0.87–0.95)	<0.001	
Model 1+ grip strength	0.95 (0.93–0.98)	0.08	0.98 (0.94–1.01)	0.20	0.91 (0.87–0.95)	<0.001	
Model 1+ gait speed	0.97 (0.94–1.00)	0.55	0.98 (0.95–1.02)	0.40	0.91 (0.87–0.95)	<0.001	
Model 1+ total fat (bioimpedance)	0.96 (0.94–0.99)	0.42	0.98 (0.95–1.02)	0.33	0.91 (0.87–0.95)	<0.001	
Model 1+ blood biomarkers	0.96 (0.93–0.98)	0.10	0.98 (0.95–1.02)	0.34	0.90 (0.86–0.94)	<0.001	
Model 2	0.96 (0.94–0.99)	0.08	0.99 (0.95–1.02)	0.52	0.91 (0.87–0.95)	<0.001	
Incident knee arthroplasty							
Unadjusted	0.82 (0.79–0.84)	<0.001	0.82 (0.78–0.85)	<0.001	0.81 (0.78–0.85)	<0.001	0.86
Model 1	0.92 (0.90–0.95)	<0.001	0.92 (0.88–0.96)	<0.001	0.93 (0.88–0.97)	0.001	
Model 1+ heel BMD	0.93 (0.90–0.96)	<0.001	0.91 (0.88–0.95)	<0.001	0.93 (0.89–0.97)	0.001	
Model 1+ grip strength	0.92 (0.89–0.95)	<0.001	0.92 (0.88–0.96)	<0.001	0.93 (0.88–0.97)	<0.001	
Model 1+ gait speed	0.94 (0.91–0.97)	<0.001	0.93 (0.89–0.97)	0.001	0.94 (0.90–0.99)	0.01	
Model 1+ total fat (bioimpedance)	0.92 (0.89–0.95)	<0.001	0.92 (0.89–0.97)	<0.001	0.94 (0.89–0.98)	0.005	
Model 1+ blood biomarkers	0.93 (0.90–0.96)	<0.001	0.93 (0.89–0.97)	<0.001	0.92 (0.88–0.97)	0.001	
Model 2	0.93 (0.91–0.96)	<0.001	0.93 (0.90–0.98)	0.002	0.94 (0.90–0.99)	0.01	

Model 1: adjusted for age, age quadratic, white cell count, ethnicity, smoking, alcohol, physical activity and menopause (women).Blood biomarkers included are: C‐reactive protein, calcium, alkaline phosphatase, urate, SHBG, creatinine, cystatin C, HbA1c.Model 2 = model 1+ heel BMD, grip strength, gait speed, total fat, blood biomarkers.

## Discussion

Our study is the largest exploration to date of telomere length associations with incident fracture and arthroplasty. We discovered modest associations between greater LTL for age and lower risk of incident fracture (predominantly in women) or arthroplasty independent of confounding factors.

Although, to our knowledge, whereas associations between LTL for age and incident fractures or arthroplasty have not been documented previously, several groups have studied the relationship between LTL and BMD or osteoarthritis. Thus, in the TwinsUK population‐based cohort of 2150 women aged 18–80 years, longer LTL (controlled for age) was positively associated with BMD at the spine and forearm and inversely associated with risk of osteoporosis.^(^ ^26^ ^)^ Two large Chinese studies have reported opposing findings using femoral neck BMD, the global standard for osteoporosis diagnosis and fracture risk assessment. In a study of 1017 elderly Chinese men and women, consistent with our findings, greater LTL was associated with greater femoral neck BMD in women under 60 years but with a declining effect size with age and no association documented in men.^(^ ^12^ ^)^ However, in a larger cohort of 1867 Chinese elderly individuals aged 65 years or older, there were no apparent associations between LTL and BMD at the total hip or femoral neck.^(^ ^15^ ^)^ A similar lack of association was observed among 460 women (mean age 63.9 years) where BMD was measured at the lumbar spine, total hip, and femoral neck.^(^ ^11^ ^)^ Other (much smaller) studies also have provided mixed results.^(^ ^17^ ^)^ We identified only one study that considered fractures, which reported that, among 2750 men and women aged 70–79 years with osteoporosis or prior fractures, there were no associations between LTL and BMD or fractures in men or women.^(^ ^27^ ^)^ In our much larger analysis, adjustment for heel eBMD did not appear to influence already small effect sizes. It is notable that the associations with fracture outcomes were weak, suggesting that the power to detect such associations in smaller cohorts will be minimal.

There is even less previous evidence linking LTL with osteoarthritis. What evidence there is generally originates from very small investigations. Shorter LTL in osteoarthritis patients (hip, *n* = 15; knee, *n* = 30) compared with healthy controls (*n* = 11) was observed in one small study.^(^ ^28^ ^)^ A similar difference, controlling for age, was documented in 160 patients with osteoarthritis at the hand versus 926 healthy controls in the TwinsUK cohort.^(^ ^29^ ^)^ In contrast, Tamayo and colleagues^(^ ^30^ ^)^ did not find differences in LTL between individuals with osteoarthritis compared with healthy individuals, although the osteoarthritis patients had longer LTL in chondrocytes compared leukocytes, a difference that was not found among the healthy individuals.

Although the observed effect sizes are very small and thus unlikely to contribute to future clinical risk assessment algorithms, they do inform understanding of aging. Evidence to date has been relatively inconsistent with regard to associations between LTL and both osteoarthritis and osteoporosis.^(^ ^17^, ^31^ ^)^ Studies have suggested that the proliferative capacity and osteogenic potential of cultured mesenchymal stromal cells from patients with osteoporosis, consistent with our findings suggesting accelerated aging.^(^ ^32^ ^)^ Differences in cell transcriptomes between patients with osteoporosis and age‐matched health subjects potentially implicate epigenetic changes,^(^ ^33^, ^34^ ^)^ although the relationships between these and telomere length is unknown. In vivo mouse models have demonstrated that telomere dysfunction can induce attenuation of osteoblast differentiation in mice, with accelerated aging.^(^ ^35^ ^)^ Consistent with the observations on bone, chondrocytes from patients with osteoarthritis appeared to develop an accelerated senescence phenotype.^(^ ^36^ ^)^ Studies of patients with knee osteoarthritis compared to healthy controls have suggested that plasma hepatocyte growth factor, vascular endothelial growth factor, and granulocyte colony‐stimulating factor are negatively associated with leukocyte telomere length,^(^ ^37^ ^)^ and may implicate oxidative stress and chronic inflammation in underlying mechanisms; indeed, further studies have suggested that higher levels of oxidative stress can accelerate telomere shortening.^(^ ^5^, ^38^ ^)^ Intriguingly, such mechanisms have been implemented in other aspects of aging, such as sarcopenia,^(^ ^39^ ^)^ and chronic inflammation associated with aging (inflammaging), is increasingly recognized as a common biological process underlying deterioration across multiple organ systems.^(^ ^40^, ^41^ ^)^

Our findings from models including adjustment for blood biomarkers including CRP are consistent with the notion that associations between telomere length and the outcomes of fracture and arthroplasty are independent of measures of inflammation (possibly counter to the studies cited above, but of course with important consideration of the entirely different investigational constructs, and acknowledgement that CRP is only one of several potential biomarkers for systemic inflammation). A further consideration is the consistency, or not, of telomere length between leucocytes and individual tissues, and whether the measure in blood cells represents a direct mechanism or indirectly reflects other processes which then influence outcome.^(^ ^17^, ^31^ ^)^ In the few studies that have compared telomere length across tissues, the results are inconsistent.^(^ ^42^, ^43^, ^44^, ^45^, ^46^ ^)^ Finally, as we have demonstrated,^(^ ^14^, ^47^, ^48^ ^)^ variation in telomere length is substantially greater between individuals of the same age and sex compared with variation over time. Although a decrease in telomere length with age is well documented,^(^ ^14^ ^)^ the magnitude of this effect appears small compared with differences between individuals, which again raises questions as to how important change in telomere length is as a key process of aging, as compared with, for example, associations with peak achieved mass and function. Certainly, the very modest effect sizes for osteoporotic fracture and arthroplasty suggest little room for therapeutic intervention or indeed for incorporation into risk assessment models. However, given existing evidence linking greater telomere length to healthier diet, lower alcohol intake, and greater physical activity,^(^ ^49^ ^)^ all of which are associated with reduced fracture risk, our results provide further evidence to support the role of a healthy lifestyle in older age,^(^ ^50^, ^51^ ^)^ together with some modest indication of associations between telomere length and fracture/arthroplasty outcomes independent of such considerations.

We studied the largest cohort to date with the gold standard measure of LTL and reliable outcome ascertainment from linkage in a prospective study. However, there are some limitations that should be considered in the interpretation of our findings. First, as with many such cohorts, there is evidence of healthy selection bias in UK Biobank compared with the general UK population. This may reduce our ability to discern associations because of reduced range of exposures or outcomes. Second, given the modest magnitude of the associations observed, and the small proportion of the cohort who are of black, Asian or minority ethnic (BAME) ethnicities, it was not possible to undertake meaningful analyses stratified by ethnicity. Third, it is very difficult to capture incident osteoarthritis, as opposed to incident arthroplasty, given the nature of the linked health data. However the case definition of osteoarthritis is variable and arthroplasty gives a much more reliable outcome, albeit defining those with the most severe disease, and of course may be influenced by access to treatment and healthcare professional practice. Furthermore, it was not possible to readily differentiate between emergency and elective arthroplasty, although it is likely that the vast majority were elective, given the codes used. Fourth, it is possible that more minor fractures, for example of the wrist, are under‐ascertained in the Hospital Episode Statistics data because these events do not usually require admission. It is possible that our findings therefore represent an underestimate of the effect size for fractures. Fifth, although we were able to account for a range of confounders, the potential for residual confounding of course always remains, and we cannot ascribe a causal relationship on the basis of these observational findings. Sixth, given the small effect sizes and small proportion of the UK Biobank population that has undergone DXA scanning at the time of this analysis, we used heel eBMD and fat mass from bioimpedance rather than the gold standard measures from DXA. Seventh, we were not able to achieve full compliance with the proportional hazards assumption for all variables considered. However, on further diagnostic investigation, it seems very unlikely that these limitations will have led to spurious associations. Finally, we were unable to measure telomere length directly in organ‐specific tissues and the relationship between LTL and that in, for example cartilage or bone, is unknown.

In conclusion, we have, to our knowledge for the first time, demonstrated modest associations between greater telomere length for age and lower risk of incident fracture or arthroplasty, independent of a wide range of confounders or potential biological mediators and with some evidence of sex specificity in the relationships. Although these findings inform our understanding of the biology of musculoskeletal aging, suggesting potential common underlying mechanisms, the small effect sizes suggest that addition of such measures into risk assessment strategies is unlikely to be a viable clinical approach.

## Author Contributions

**Elizabeth M Curtis:** Conceptualization; investigation; methodology; project administration; writing – original draft; writing – review and editing. **Veryan Codd:** Data curation; formal analysis; funding acquisition; investigation; writing – original draft; writing – review and editing. **Christopher Nelson:** Data curation; formal analysis; funding acquisition; investigation; methodology; writing – original draft; writing – review and editing. **Stefania D'Angelo:** Data curation; formal analysis; methodology; writing – original draft; writing – review and editing. **Qingning Wang:** Data curation; formal analysis; methodology; writing – review and editing. **Elias Allara:** Data curation; formal analysis; methodology; writing – review and editing. **Stephen Kaptoge:** Conceptualization; data curation; investigation; writing – review and editing. **Paul M Matthews:** Conceptualization; funding acquisition; writing – review and editing. **Jonathan H Tobias:** Conceptualization; funding acquisition; investigation; methodology; writing – review and editing. **John Danesh:** Conceptualization; formal analysis; funding acquisition; writing – review and editing. **Cyrus Cooper:** Conceptualization; data curation; funding acquisition; project administration; supervision; writing – review and editing. **Nilesh J Samani:** Conceptualization; data curation; funding acquisition; investigation; project administration; supervision; writing – review and editing. **Nicholas C Harvey:** Conceptualization; formal analysis; funding acquisition; methodology; project administration; supervision; writing – original draft; writing – review and editing.

## Conflicts of interest

All authors have no disclosures in relation to this manuscript.

## Supporting information


**Appendix S1** Supplementary Information
Fig. S1
Fig. S2
Table S1
Table S2
Table S3
Table S4
Table S5
Table S6
Table S7
Table S8
Table S9

## Data Availability

All data are available via an access application to UK Biobank. https://www.ukbiobank.ac.uk/enable-your-research
