# Supplementary material for: Telomere Length and Risk of Incident Fracture and Arthroplasty: Findings From UK Biobank
Source: J Bone Miner Res. 2022 Sep 13;37(10):1997–2004. doi: 10.1002/jbmr.4664 (PMC9826022; doi:10.1002/jbmr.4664)

**Greater telomere length for age is associated with lower risk of incident fracture and arthroplasty: findings from UK Biobank**

Elizabeth M Curtis^1^*, Veryan Codd^2,3^*, Christopher Nelson^2,3^*, Stefania D’Angelo^1^*, Qingning Wang^2,3^, Elias Allara,^7,8,9^, Stephen Kaptoge^7,8,9^, Paul Matthews^4^, Jon Tobias^5,6^, John Danesh^7-11^, Cyrus Cooper^1,12,13+^, Nilesh J Samani^2,3+^, Nicholas C Harvey^1,12+^

**Online supplementary material**

**Supplementary Table 1:** Hospital Episode Statistics and Operating Procedure Codes

| **Outcome** | **ICD Codes** |
| --- | --- |
| Any fracture, HES | 7218, 7330, 7331, 8000, 8001, 8002, 8003, 8010, 8011, 8012, 8013, 8020, 8021, 8026, 8027, 8024, 8025, 8736, 8737, 8022, 8023, 8040, 8041, 8042, 8043, 8028, 8029, 8030, 8031, 8032, 8033, 8050, 8051, 8060, 8061, 8075, 8076, 8052, 8053, 8062, 8063, 8072, 8073, 8070, 8071, 8074, 8090, 8091, 8054, 8055, 8064, 8065, 8056, 8057, 8066, 8067, 8084, 8085, 8080, 8081, 8082, 8083, 8088, 8089, 8100, 8101, 8110, 8111, 8120, 8121, 8122, 8123, 8124, 8125, 8180, 8181, 8130, 8131, 8132, 8133, 8134, 8135, 8140, 8141, 8150, 8151, 8160, 8161, 8170, 8171, 8200, 8201, 8208, 8209, 8202, 8203, 8210, 8211, 8212, 8213, 8220, 8221, 8250, 8251, 8252, 8253, 8260, 8261, 8270, 8271, 8190, 8191, 8280, 8281, 8058, 8059, 8068, 8069, 8290, 8291, 8230, 8231, 8232, 8233, 8248, 8249, 8240, 8241, 8242, 8243, 8244, 8245, 8246, 8247, M484, M4840, M4844, M4845, M4846, M4849, M485, M4850, M4852, M4854, M4855, M4856, M4857, M4859, M800, M8000, M8002, M8003, M8005, M8006, M8007, M8008, M8009, M801, M8019, M802, M8025, M803, M804, M8040, M8045, M8048, M8049, M805, M8050, M8055, M8057, M8058, M8059, M808, M8080, M8081, M8088, M8089, M809, M8090, M8091, M8093, M8095, M8097, M8098, M8099, S020, S0200, S0201, S021, S0210, S0211, S022, S0220, S0221, S023, S0230, S0231, S024, S0240, S0241, S025, S0250, S026, S0260, S0261, S027, S0270, S0271, S028, S0280, S029, S0290, S0291, S120, S1200, S1201, S121, S1210, S1211, S122, S1220, S1221, S127, S1270, S128, S1280, S129, S1290, S220, S2200, S2201, S221, S2210, S2211, S222, S2220, S223, S2230, S224, S2240, S225, S2250, S228, S2280, S229, S320, S3200, S3201, S321, S3210, S322, S3220, S323, S3230, S324, S3240, S3241, S325, S3250, S327, S3270, S3271, S328, S3280, S3281, S420, S4200, S4201, S421, S4210, S4211, S422, S4220, S4221, S423, S4230, S4231, S424, S4240, S4241, S427, S4270, S4271, S428, S4280, S429, S4290, S4291, S520, S5200, S5201, S521, S5210, S5211, S522, S5220, S5221, S523, S5230, S5231, S524, S5240, S5241, S525, S5250, S5251, S526, S5260, S5261, S527, S5270, S5271, S528, S5280, S5281, S529, S5290, S5291, S620, S6200, S6201, S621, S6210, S6211, S622, S6220, S6221, S623, S6230, S6231, S624, S6240, S6241, S625, S6250, S6251, S626, S6260, S6261, S627, S6270, S6271, S628, S6280, S6281, S720, S7200, S7201, S721, S7210, S7211, S722, S7220, S7221, S723, S7230, S7231, S724, S7240, S7241, S727, S7270, S7271, S728, S7280, S7281, S729, S7290, S7291, S820, S8200, S8201, S920, S9200, S9201, S921, S9210, S9211, S922, S9220, S9221, S923, S9230, S9231, S924, S9240, S9241, S925, S9250, S9251, S927, S9270, S9271, S929, S9290, T020,, T021, T0210, T022, T023, T0230, T024, T0240, T025, T0250, T026, T0260, T027, T0270, T0271, T028, T0280, T029, T08 , T0890, T10 , T1090, T1091, T12 , T140, T141, T142, T143, T144, T145, T146, T148, T149, S821, S8210, S8211, S822, S8220, S8221, S823, S8230, S8231, S824, S8240, S8241, S825, S8250, S8251, S826, S8260, S8261, S827, S8270, S8271, S828, S8280, S8281, S829, S8290 |
| Osteoporotic fracture; HES | 7331, 8050, 8051, 8060, 8061, 8075, 8076, 8052, 8053, 8062, 8063, 8072, 8073, 8070, 8071, 8074, 8090, 8091, 8054, 8055, 8064, 8065, 8056, 8057, 8066, 8067, 8084, 8085, 8080, 8081, 8082, 8083, 8088, 8089, 8054, 8055, 8064, 8065, 8084, 8085, 8088, 8089, 8100, 8101, 8120, 8121, 8122, 8123, 8124, 8125, 8180, 8181, 8120, 8121, 8124, 8125, 8130, 8131, 8130, 8131, 8132, 8133, 8134, 8135, 8130, 8131, 8134, 8135, 8180, 8181, 8200, 8201, 8208, 8209, 8202, 8203, 8210, 8211, 8212, 8213, 8090, 8091, 8058, 8059, 8068, 8069, M800, M8000, M8002, M8003, M8005, M8006, M8007, M8008, M8009, M801, M8019, M802, M8025, M803, M804, M8040, M8045, M8048, M8049, M805, M8050, M8055, M8057, M8058, M8059, M808, M8080, M8081, M8088, M8089, M809, M8090, M8091, M8093, M8095, M8097, M8098, M8099, S120, S1200, S1201, S121, S1210, S1211, S122, S1220, S1221, S127, S1270, S128, S1280, S129, S1290, S220, S2200, S2201, S221, S2210, S2211, S222, S2220, S223, S2230, S224, S2240, S225, S2250, S228, S2280, S229, S320, S3200, S3201, S321, S3210, S322, S3220, S323, S3230, S324, S3240, S3241, S325, S3250, S327, S3270, S3271, S328, S3280, S3281, S420, S4200, S4201, S421, S4210, S4211, S422, S4220, S4221, S423, S4230, S4231, S424, S4240, S4241, S427, S4270, S4271, S428, S4280, S429, S4290, S4291, S520, S5200, S5201, S521, S5210, S5211, S522, S5220, S5221, S523, S5230, S5231, S524, S5240, S5241, S525, S5250, S5251, S526, S5260, S5261, S527, S5270, S5271, S528, S5280, S5281, S529, S5290, S5291, S720, S7200, S7201, S721, S7210, S7211, S722, S7220, S7221, S723, S7230, S7231, S724, S7240, S7241, S727, S7270, S7271, S728, S7280, S7281, S729, S7290, S7291, T021, T08 , T0890 |
| Hip arthroplasty (primary); OPCS | W371, W372, W379, W381, W382, W389, W391, W392, W399, W931, W932, W939, W941, W942, W949, W951, W952, W959, W581, W582, W589, W521, W529, W529, W531, W532, W539, W541, W542, W549 |
| Hip arthroplasty (secondary); OPCS | W37, W373, W374, W378, W370, W38, W383, W384, W388, W380, W39, W393, W395, W398, W390, W93, W933, W938, W930, W94, W943, W948, W940, W95, W953, W954, W958, W950, W58, W588, W580, W52, W523, W528, W520, W53, W533, W538, W530, W54, W543, W548, W540 |
| Knee arthroplasty (primary); OPCS | W401, W402, W409, W411, W412, W419, W421, W422, W429, W521, W522, W529, W531, W532, W539, W541, W542, W549, W581, W582, W589 |
| Knee arthroplasty (secondary); OPCS | W40, W403, W404, W408, W400, W41, W413, W414, W418, W410, W42, W423, W428, W420, W425, W52, W523, W528, W520, W53, W533, W538, W530, W54, W543, W548, W540, W58, W588, W580 |

**Supplementary Table 2:** Associations between LTL and blood biomarkers, adjusted for age

|  | **Women** | | | **Men** | | | p sex interaction |
| --- | --- | --- | --- | --- | --- | --- | --- |
|  | n | β (95% CI) | p | n | β (95% CI) | p |  |
| C-reactive protein | 243745 | **-0.041 ( -0.045, -0.037)** | <0.001 | 205927 | **-0.030( -0.034, -0.026)** | <0.001 | <0.001 |
| 25(OH)-vitamin D | 230273 | 0.001( -0.003, 0.005) | 0.61 | 199995 | -0.001( -0.005, 0.004) | 0.68 | <0.001 |
| Calcium | 221853 | -0.001 ( -0.005, 0.003) | 0.60 | 190678 | -0.004( -0.008, 0.001) | 0.10 | <0.001 |
| Alkaline phosphatase | 244190 | **-0.012 ( -0.016, -0.008)** | <0.001 | 206468 | **-0.007( -0.011, -0.002)** | 0.002 | <0.001 |
| Urate | 243862 | **-0.028( -0.032, -0.025)** | <0.001 | 206236 | **-0.019( -0.022, -0.015)** | <0.001 | <0.001 |
| SHBG | 219429 | -0.001 ( -0.005, 0.003) | 0.56 | 189269 | **-0.029( -0.032, -0.025)** | <0.001 | <0.001 |
| Oestradiol | 55765 | **-0.008( -0.016, -0.001)** | 0.03 | 17760 | -0.006( -0.016, 0.004) | 0.26 | <0.001 |
| Creatinine | 244056 | **-0.011( -0.014, -0.008)** | <0.001 | 206362 | -0.003( -0.006, 0.000) | 0.09 | 0.005 |
| Phosphate | 221525 | 0.001( -0.003, 0.005) | 0.67 | 190390 | 0.001( -0.004, 0.005) | 0.76 | <0.001 |
| Cystatin C | 244158 | **-0.040( -0.044, -0.036)** | <0.001 | 206454 | **-0.032 ( -0.036, -0.028)** | <0.001 | <0.001 |
| HbA1c | 243716 | **-0.005( -0.009, -0.002)** | 0.004 | 205872 | **-0.019( -0.023, -0.014)** | <0.001 | 0.01 |

Bold type indicates associations are 95% CI exclude unity.

**Supplementary Table 3:** Associations between LTL and incident fractures, using largest sample size for each test.

|  |  | All |  | Women | | | Men | | | p sex interaction |
| --- | --- | --- | --- | --- | --- | --- | --- | --- | --- | --- |
|  | n | HR (95%CI) | p | n | HR (95%CI) | p | n | HR (95%CI) | p |  |
| **Any incident fracture** |  |  |  |  |  |  |  |  |  |  |
| *Unadjusted* | 472286 | 0.92 (0.91,0.93) | <0.001 | 256048 | 0.88 (0.87,0.89) | <0.001 | 216238 | 0.94 (0.92,0.96) | <0.001 | <0.001 |
| *Model 1* | 430741 | 0.98 (0.97,0.99) | 0.004 | 231558 | 0.95 (0.93,0.97) | <0.001 | 198974 | 0.98 (0.96,1.00) | 0.04 |  |
| *Model 1+ heel BMD* | 141338 | 0.99 (0.96,1.01) | 0.25 | 75941 | 0.97 (0.94,1.00) | 0.03 | 65324 | 0.99 (0.95,1.03) | 0.64 |  |
| *Model 1+ grip strength* | 429728 | 0.97 (0.96,0.98) | <0.001 | 230986 | 0.95 (0.94,0.97) | <0.001 | 198537 | 0.98 (0.96,1.00) | 0.06 |  |
| *Model 1 + gait speed* | 428513 | 0.98 (0.97,1.00) | 0.02 | 230406 | 0.95 (0.94,0.97) | <0.001 | 197905 | 0.98 (0.96,1.01) | 0.16 |  |
| *Model 1 + total fat (bioimpedance)* | 422737 | 0.98 (0.97,0.99) | 0.007 | 227813 | 0.95 (0.93,0.97) | <0.001 | 194727 | 0.98 (0.96,1.00) | 0.09 |  |
| *Model 1 + blood biomarkers* | 355057 | 0.97 (0.95,0.98) | <0.001 | 189145 | 0.95 (0.93,0.97) | <0.001 | 165740 | 0.98 (0.96,1.00) | 0.09 |  |
| *Model 2* | 111452 | 0.98 (0.95,1.00) | 0.07 | 59500 | 0.97 (0.94,1.01) | 0.10 | 51895 | 0.99 (0.94,1.03) | 0.53 |  |
| **Any incident osteoporotic fracture** |  |  |  |  |  |  |  |  |  |  |
| *Unadjusted* | 472286 | 0.90 (0.89,0.92) | <0.001 | 256048 | 0.87 (0.85,0.89) | <0.001 | 216238 | 0.91 (0.88,0.93) | <0.001 | 0.01 |
| *Model 1* | 430741 | 0.98 (0.97,1.00) | 0.04 | 231558 | 0.95 (0.93,0.97) | <0.001 | 198974 | 0.97 (0.94,1.00) | 0.02 |  |
| *Model 1+ heel BMD* | 141338 | 0.98 (0.96,1.01) | 0.25 | 75941 | 0.96 (0.93,1.00) | 0.05 | 65324 | 0.98 (0.94,1.03) | 0.53 |  |
| *Model 1+ grip strength* | 429728 | 0.97 (0.95,0.98) | <0.001 | 230986 | 0.96 (0.94,0.98) | <0.001 | 198537 | 0.97 (0.94,1.00) | 0.03 |  |
| *Model 1 + gait speed* | 428513 | 0.99 (0.97,1.00) | 0.14 | 230406 | 0.96 (0.94,0.98) | <0.001 | 197905 | 0.98 (0.95,1.00) | 0.11 |  |
| *Model 1 + total fat (bioimpedance)* | 422737 | 0.99 (0.97,1.00) | 0.09 | 227813 | 0.95 (0.93,0.97) | <0.001 | 194727 | 0.98 (0.95,1.00) | 0.09 |  |
| *Model 1 + blood biomarkers* | 355057 | 0.96 (0.95,0.98) | <0.001 | 189145 | 0.95 (0.93,0.97) | <0.001 | 165740 | 0.97 (0.94,1.00) | 0.04 |  |
| *Model 2* | 111452 | 0.97 (0.94,1.00) | 0.07 | 59500 | 0.96 (0.93,1.00) | 0.08 | 51895 | 0.98 (0.93,1.04) | 0.58 |  |

Model 1: adjusted for age, white cell count, ethnicity, smoking, alcohol, physical activity and menopause (women).

Blood biomarkers included are: C-reactive protein, calcium, alkaline phosphatase, urate, SHBG, creatinine, cystatin C, HbA1c

Model 2 = model 1+ heel BMD, grip strength, gait speed, total fat, blood biomarkers.

**Supplementary Table 4:** Associations between LTL and incident arthroplasty, using largest sample size for each test.

|  |  | All |  | Women | | | Men | | | p sex interaction |
| --- | --- | --- | --- | --- | --- | --- | --- | --- | --- | --- |
|  | n | HR (95%CI) | p | n | HR (95%CI) | p | n | HR (95%CI) | p |  |
| **Incident hip arthroplasty** |  |  |  |  |  |  |  |  |  |  |
| *Unadjusted* | 472286 | 0.88 (0.87,0.89) | <0.001 | 256048 | 0.87 (0.86,0.89) | <0.001 | 216238 | 0.87 (0.85,0.89) | <0.001 | 0.77 |
| *Model 1* | 430741 | 0.99 (0.98,1.01) | 0.22 | 231558 | 0.98 (0.96,0.99) | 0.008 | 198974 | 0.98 (0.95,1.00) | 0.03 |  |
| *Model 1+ heel BMD* | 141338 | 0.97 (0.95,1.00) | 0.04 | 75941 | 0.98 (0.95,1.01) | 0.21 | 65324 | 0.93 (0.89,0.96) | <0.001 |  |
| *Model 1+ grip strength* | 429728 | 0.98 (0.97,1.00) | 0.02 | 230986 | 0.98 (0.96,0.99) | 0.01 | 198537 | 0.98 (0.95,1.00) | 0.03 |  |
| *Model 1 + gait speed* | 428513 | 1.00 (0.99,1.01) | 0.91 | 230406 | 0.98 (0.97,1.00) | 0.09 | 197905 | 0.98 (0.96,1.01) | 0.16 |  |
| *Model 1 + total fat (bioimpedance)* | 422737 | 0.99 (0.98,1.00) | 0.21 | 227813 | 0.98 (0.96,1.00) | 0.04 | 194727 | 0.98 (0.96,1.00) | 0.09 |  |
| *Model 1 + blood biomarkers* | 355057 | 0.98 (0.97,1.00) | 0.02 | 189145 | 0.98 (0.96,0.99) | 0.01 | 165740 | 0.97 (0.95,0.99) | 0.009 |  |
| *Model 2* | 111452 | 0.96 (0.94,0.99) | 0.009 | 59500 | 0.99 (0.95,1.03) | 0.56 | 51895 | 0.91 (0.88,0.96) | <0.001 |  |
| **Incident knee arthroplasty** |  |  |  |  |  |  |  |  |  |  |
| *Unadjusted* | 472286 | 0.85 (0.84,0.87) | <0.001 | 256048 | 0.85 (0.84,0.87) | <0.001 | 216238 | 0.85 (0.83,0.87) | <0.001 | 0.67 |
| *Model 1* | 430741 | 0.96 (0.95,0.98) | <0.001 | 231558 | 0.95 (0.93,0.87) | <0.001 | 198974 | 0.96 (0.94,0.98) | 0.001 |  |
| *Model 1+ heel BMD* | 141338 | 0.94 (0.91,0.96) | <0.001 | 75941 | 0.93 (0.89,0.96) | <0.001 | 65324 | 0.93 (0.89,0.97) | 0.001 |  |
| *Model 1+ grip strength* | 429728 | 0.96 (0.94,0.97) | <0.001 | 230986 | 0.96 (0.94,0.98) | <0.001 | 198537 | 0.96 (0.94,0.98) | 0.001 |  |
| *Model 1 + gait speed* | 428513 | 0.98 (0.96,0.99) | 0.001 | 230406 | 0.96 (0.94,0.98) | 0.001 | 197905 | 0.98 (0.95,1.00) | 0.05 |  |
| *Model 1 + total fat (bioimpedance)* | 422737 | 0.96 (0.94,0.97) | <0.001 | 227813 | 0.96 (0.94,0.98) | <0.001 | 194727 | 0.97 (0.95,0.99) | 0.01 |  |
| *Model 1 + blood biomarkers* | 355057 | 0.96 (0.95,0.98) | <0.001 | 189145 | 0.96 (0.94,0.98) | 0.001 | 165740 | 0.95 (0.93,0.98) | <0.001 |  |
| *Model 2* | 111452 | 0.94 (0.91,0.97) | <0.001 | 59500 | 0.94 (0.90,0.98) | 0.002 | 51895 | 0.94 (0.90,0.99) | 0.01 |  |

Model 1: adjusted for age, white cell count, ethnicity, smoking, alcohol, physical activity and menopause (women).

Blood biomarkers included are: C-reactive protein, calcium, alkaline phosphatase, urate, SHBG, creatinine, cystatin C, HbA1c

Model 2 = model 1+ heel BMD, grip strength, gait speed, total fat, blood biomarkers.

**Supplementary Table 5:** Associations between LTL and incident primary arthroplasty

|  |  | All |  | Women | | | Men | | | p sex interaction |
| --- | --- | --- | --- | --- | --- | --- | --- | --- | --- | --- |
|  | n | HR (95%CI) | p | n | HR (95%CI) | p | n | HR (95%CI) | p |  |
| **Incident hip arthroplasty** |  |  |  |  |  |  |  |  |  |  |
| *Unadjusted* | 472286 | 0.88 (0.87,0.90) | <0.001 | 256048 | 0.87 (0.86,0.89) | <0.001 | 216238 | 0.87 (0.85,0.89) | <0.001 | 0.72 |
| *Model 1* | 430741 | 0.99 (0.98,1.01) | 0.22 | 231558 | 0.98 (0.96,1.00) | 0.01 | 198974 | 0.97 (0.95,0.99) | 0.02 |  |
| *Model 1+ heel BMD* | 141338 | 0.97 (0.95,1.00) | 0.03 | 75941 | 0.98 (0.95,1.01) | 0.20 | 65324 | 0.92 (0.89,0.96) | <0.001 |  |
| *Model 1+ grip strength* | 429728 | 0.98 (0.97,1.00) | 0.03 | 230986 | 0.98 (0.96,1.00) | 0.02 | 198537 | 0.97 (0.95,0.99) | 0.02 |  |
| *Model 1 + gait speed* | 428513 | 1.00 (0.99,1.01) | 0.98 | 230406 | 0.99 (0.97,1.00) | 0.11 | 197905 | 0.98 (0.96,1.00) | 0.09 |  |
| *Model 1 + total fat (bioimpedance)* | 422737 | 0.99 (0.98,1.01) | 0.21 | 227813 | 0.98 (0.96,1.00) | 0.06 | 194727 | 0.98 (0.96,1.00) | 0.06 |  |
| *Model 1 + blood biomarkers* | 355057 | 0.98 (0.97,1.00) | 0.02 | 189145 | 0.98 (0.96,0.99) | 0.01 | 165740 | 0.97 (0.94,0.99) | 0.006 |  |
| *Model 2* | 111452 | 0.96 (0.94,0.99) | 0.008 | 59500 | 0.99 (0.95,1.03) | 0.54 | 51895 | 0.91 (0.87,0.96) | <0.001 |  |
| **Incident knee arthroplasty** |  |  |  |  |  |  |  |  |  |  |
| *Unadjusted* | 472286 | 0.85 (0.84,0.87) | <0.001 | 256048 | 0.85 (0.84,0.87) | <0.001 | 216238 | 0.85 (0.83,0.87) | <0.001 | 0.78 |
| *Model 1* | 430741 | 0.96 (0.95,0.98) | <0.001 | 231558 | 0.95 (0.93,0.97) | <0.001 | 198974 | 0.96 (0.94,0.99) | 0.002 |  |
| *Model 1+ heel BMD* | 141338 | 0.94 (0.91,0.96) | <0.001 | 75941 | 0.93 (0.89,0.96) | <0.001 | 65324 | 0.93 (0.89,0.97) | 0.001 |  |
| *Model 1+ grip strength* | 429728 | 0.96 (0.94,0.97) | <0.001 | 230986 | 0.96 (0.94,0.98) | <0.001 | 198537 | 0.96 (0.94,0.99) | 0.002 |  |
| *Model 1 + gait speed* | 428513 | 0.98 (0.96,0.99) | 0.002 | 230406 | 0.96 (0.94,0.98) | 0.001 | 197905 | 0.98 (0.96,1.00) | 0.07 |  |
| *Model 1 + total fat (bioimpedance)* | 422737 | 0.96 (0.94,0.98) | <0.001 | 227813 | 0.96 (0.94,0.98) | <0.001 | 194727 | 0.97 (0.95,1.00) | 0.02 |  |
| *Model 1 + blood biomarkers* | 355057 | 0.96 (0.95,0.98) | <0.001 | 189145 | 0.96 (0.94,0.98) | 0.001 | 165740 | 0.96 (0.93,0.98) | 0.001 |  |
| *Model 2* | 111452 | 0.94 (0.91,0.97) | <0.001 | 59500 | 0.94 (0.90,0.98) | 0.003 | 51895 | 0.94 (0.90,0.99) | 0.02 |  |

Model 1: adjusted for age, white cell count, ethnicity, smoking, alcohol, physical activity and menopause (women); Blood biomarkers included are: C-reactive protein, calcium, alkaline phosphatase, urate, SHBG, creatinine, cystatin C, HbA1c; Model 2 = model 1+ heel BMD, grip strength, gait speed, total fat, blood biomarkers.

**Supplementary Table 6:** Associations between LTL and incident fractures, with adjustment for LTL regression dilution ratio.

|  |  | All |  | Women | | | Men | | | p sex interaction |
| --- | --- | --- | --- | --- | --- | --- | --- | --- | --- | --- |
|  | n | HR (95%CI) | p | n | HR (95%CI) | p | n | HR (95%CI) | p |  |
| **Any incident fracture** |  |  |  |  |  |  |  |  |  |  |
| *Unadjusted* | 472286 | 0.88 (0.87,0.90) | <0.001 | 256048 | 0.83 (0.81,0.85) | <0.001 | 216238 | 0.92 (0.89,0.94) | <0.001 | <0.001 |
| *Model 1* | 430741 | 0.97 (0.95,0.99) | 0.004 | 231558 | 0.93 (0.91,0.95) | <0.001 | 198974 | 0.97 (0.94,1.00) | 0.04 |  |
| *Model 1+ heel BMD* | 141338 | 0.98 (0.95,1.01) | 0.25 | 75941 | 0.95 (0.91,0.99) | 0.03 | 65324 | 0.99 (0.96,1.04) | 0.64 |  |
| *Model 1+ grip strength* | 429728 | 0.95 (0.94,0.97) | <0.001 | 230986 | 0.93 (0.91,0.96) | <0.001 | 198537 | 0.97 (0.94,1.00) | 0.06 |  |
| *Model 1 + gait speed* | 428513 | 0.98 (0.96,1.00) | 0.02 | 230406 | 0.93 (0.91,0.96) | <0.001 | 197905 | 0.98 (0.95,1.01) | 0.16 |  |
| *Model 1 + total fat (bioimpedance)* | 422737 | 0.97 (0.95,0.99) | 0.007 | 227813 | 0.93 (0.90,0.95) | <0.001 | 194727 | 0.97 (0.94,1.00) | 0.09 |  |
| *Model 1 + blood biomarkers* | 355057 | 0.95 (0.93,0.97) | <0.001 | 189145 | 0.93 (0.91,0.96) | <0.001 | 165740 | 0.97 (0.94,1.00) | 0.09 |  |
| *Model 2* | 111452 | 0.96 (0.93,1.00) | 0.07 | 59500 | 0.96 (0.91,1.01) | 0.10 | 51895 | 0.98 (0.92,1.04) | 0.53 |  |
| **Any incident osteoporotic fracture** |  |  |  |  |  |  |  |  |  |  |
| *Unadjusted* | 472286 | 0.86 (0.84,0.88) | <0.001 | 256048 | 0.82 (0.79,0.84) | <0.001 | 216238 | 0.87 (0.83,0.90) | <0.001 | 0.01 |
| *Model 1* | 430741 | 0.97 (0.95,1.00) | 0.04 | 231558 | 0.93 (0.90,0.96) | <0.001 | 198974 | 0.95 (0.91,0.99) | 0.02 |  |
| *Model 1+ heel BMD* | 141338 | 0.98 (0.94,1.02) | 0.25 | 75941 | 0.95 (0.90,1.00) | 0.05 | 65324 | 0.98 (0.91,1.05) | 0.53 |  |
| *Model 1+ grip strength* | 429728 | 0.95 (0.93,0.98) | <0.001 | 230986 | 0.94 (0.91,0.97) | <0.001 | 198537 | 0.96 (0.92,1.00) | 0.03 |  |
| *Model 1 + gait speed* | 428513 | 0.98 (0.96,1.01) | 0.14 | 230406 | 0.94 (0.91,0.96) | <0.001 | 197905 | 0.97 (0.93,1.01) | 0.11 |  |
| *Model 1 + total fat (bioimpedance)* | 422737 | 0.98 (0.96,1.00) | 0.09 | 227813 | 0.93 (0.90,0.96) | <0.001 | 194727 | 0.96 (0.92,1.01) | 0.09 |  |
| *Model 1 + blood biomarkers* | 355057 | 0.95 (0.92,0.97) | <0.001 | 189145 | 0.93 (0.90,0.96) | <0.001 | 165740 | 0.95 (0.91,1.00) | 0.04 |  |
| *Model 2* | 111452 | 0.96 (0.91,1.00) | 0.07 | 59500 | 0.95 (0.89,1.01) | 0.08 | 51895 | 0.98 (0.90,1.06) | 0.58 |  |

Model 1: adjusted for age, white cell count, ethnicity, smoking, alcohol, physical activity and menopause (women).

Blood biomarkers included are: C-reactive protein, calcium, alkaline phosphatase, urate, SHBG, creatinine, cystatin C, HbA1c

Model 2 = model 1+ heel BMD, grip strength, gait speed, total fat, blood biomarkers.

**Supplementary Table 7:** Associations between LTL and incident arthroplasty, with adjustment for LTL regression dilution ratio.

|  |  | All |  | Women | | | Men | | | p sex interaction |
| --- | --- | --- | --- | --- | --- | --- | --- | --- | --- | --- |
|  | n | HR (95%CI) | p | n | HR (95%CI) | p | n | HR (95%CI) | p |  |
| **Incident hip arthroplasty** |  |  |  |  |  |  |  |  |  |  |
| *Unadjusted* | 472286 | 0.83 (0.82,0.85) | <0.001 | 256048 | 0.82 (0.80,0.84) | <0.001 | 216238 | 0.81 (0.79,0.84) | <0.001 | 0.77 |
| *Model 1* | 430741 | 0.99 (0.97,1.01) | 0.22 | 231558 | 0.97 (0.94,0.99) | 0.008 | 198974 | 0.96 (0.93,1.00) | 0.03 |  |
| *Model 1+ heel BMD* | 141338 | 0.96 (0.93,1.00) | 0.04 | 75941 | 0.97 (0.93,1.02) | 0.21 | 65324 | 0.89 (0.84,0.94) | <0.001 |  |
| *Model 1+ grip strength* | 429728 | 0.98 (0.96,1.00) | 0.02 | 230986 | 0.97 (0.94,0.99) | 0.01 | 198537 | 0.96 (0.93,1.00) | 0.03 |  |
| *Model 1 + gait speed* | 428513 | 1.00 (0.98,1.02) | 0.91 | 230406 | 0.98 (0.95,1.00) | 0.09 | 197905 | 0.98 (0.95,1.01) | 0.16 |  |
| *Model 1 + total fat (bioimpedance)* | 422737 | 0.99 (0.97,1.01) | 0.21 | 227813 | 0.97 (0.95,1.00) | 0.04 | 194727 | 0.97 (0.94,1.00) | 0.09 |  |
| *Model 1 + blood biomarkers* | 355057 | 0.97 (0.95,0.99) | 0.02 | 189145 | 0.93 (0.94,0.99) | 0.01 | 165740 | 0.95 (0.92,0.99) | 0.009 |  |
| *Model 2* | 111452 | 0.95 (0.91,0.99) | 0.009 | 59500 | 0.98 (0.93,1.04) | 0.56 | 51895 | 0.89 (0.82,0.93) | <0.001 |  |
| **Incident knee arthroplasty** |  |  |  |  |  |  |  |  |  |  |
| *Unadjusted* | 472286 | 0.79 (0.78,0.81) | <0.001 | 256048 | 0.79 (0.77,0.81) | <0.001 | 216238 | 0.79 (0.76,0.81) | <0.001 | 0.67 |
| *Model 1* | 430741 | 0.94 (0.92,0.96) | <0.001 | 231558 | 0.93 (0.90,0.96) | <0.001 | 198974 | 0.94 (0.91,0.98) | 0.001 |  |
| *Model 1+ heel BMD* | 141338 | 0.91 (0.87,0.95) | <0.001 | 75941 | 0.90 (0.85,0.95) | <0.001 | 65324 | 0.90 (0.85,0.96) | 0.001 |  |
| *Model 1+ grip strength* | 429728 | 0.93 (0.91,0.96) | <0.001 | 230986 | 0.93 (0.91,0.96) | <0.001 | 198537 | 0.94 (0.91,0.98) | 0.001 |  |
| *Model 1 + gait speed* | 428513 | 0.96 (0.94,0.99) | 0.001 | 230406 | 0.95 (0.92,0.98) | 0.001 | 197905 | 0.97 (0.93,1.00) | 0.05 |  |
| *Model 1 + total fat (bioimpedance)* | 422737 | 0.94,0.92,0.96 | <0.001 | 227813 | 0.95 (0.92,0.98) | <0.001 | 194727 | 0.96 (0.92,0.99) | 0.01 |  |
| *Model 1 + blood biomarkers* | 355057 | 0.94 (0.92,0.97) | <0.001 | 189145 | 0.94 (0.91,0.98) | 0.001 | 165740 | 0.93 (0.90,0.97) | <0.001 |  |
| *Model 2* | 111452 | 0.91 (0.87,0.95) | <0.001 | 59500 | 0.91 (0.85,0.97) | 0.002 | 51895 | 0.92 (0.86,0.98) | 0.01 |  |

Model 1: adjusted for age, white cell count, ethnicity, smoking, alcohol, physical activity and menopause (women).

Blood biomarkers included are: C-reactive protein, calcium, alkaline phosphatase, urate, SHBG, creatinine, cystatin C, HbA1c

Model 2 = model 1+ heel BMD, grip strength, gait speed, total fat, blood biomarkers.

**Supplementary Table 8:** Model 1 analyses replicated within each quarter of LTL.

|  | **Cohort with complete data** | | | | | |
| --- | --- | --- | --- | --- | --- | --- |
|  | Women | | | Men | | |
|  | n | HR (95%CI) | p | n | HR (95%CI) | p |
| **Osteoporotic fracture** |  |  |  |  |  |  |
| Q1 (-15.3,-0.65) | 13280 | 0.93 (0.80,1.07) | 0.31 | 14859 | 0.97 (0.81,1.15) | 0.72 |
| Q2 (-0.65,-0.002) | 14495 | 0.80 (0.53,1.21) | 0.29 | 13483 | 0.74 (0.73,1.29) | 0.29 |
| Q3 (-0.002,0.65) | 15250 | 0.97 (0.63,1.51) | 0.90 | 12499 | 0.95 (0.52,1.74) | 0.87 |
| Q4 (0.65,12.1) | 16475 | 1.11 (0.96,1.28) | 0.16 | 11054 | 0.89 (0.71,1.13) | 0.35 |
|  |  |  |  |  |  |  |
| **Hip arthroplasty** |  |  |  |  |  |  |
| Q1 (-15.3,-0.65) | 13280 | 0.94 (0.82,1.07) | 0.33 | 14859 | 0.91 (0.81,1.03) | 0.15 |
| Q2 (-0.65,-0.002) | 14495 | 0.84 (0.58,1.22) | 0.36 | 13483 | 0.72 (0.46,1.12) | 0.15 |
| Q3 (-0.002,0.65) | 15250 | 1.34 (0.91,1.97) | 0.14 | 12499 | 1.31 (0.79,2.14) | 0.29 |
| Q4 (0.65,12.1) | 16475 | 0.99 (0.87,1.13) | 0.87 | 11054 | 1.01 (0.84,1.22) | 0.91 |
|  |  |  |  |  |  |  |
| **Knee** **arthroplasty** |  |  |  |  |  |  |
| Q1 (-15.3,-0.65) | 13280 | 0.88 (0.76,1.01) | 0.07 | 14859 | 0.85 (0.75,0.96) | 0.009 |
| Q2 (-0.65,-0.002) | 14495 | 0.73 (0.47,1.12) | 0.15 | 13483 | 1.10 (0.69,1.75) | 0.69 |
| Q3 (-0.002,0.65) | 15250 | 1.13 (0.70,1.81) | 0.62 | 12499 | 1.13 (0.73,2.06) | 0.43 |
| Q4 (0.65,12.1) | 16475 | 1.05 (0.90,1.24) | 0.53 | 11054 | 1.08 (0.89,1.31) | 0.43 |

**Supplementary Table 9:** Model 1 analyses stratified according to covariates in models which did not meet proportional hazards assumption.

|  | **Cohort with complete data** | | | | | |
| --- | --- | --- | --- | --- | --- | --- |
|  | Women | | | Men | | |
|  | n | HR (95%CI) | p | n | HR (95%CI) | p |
| **Osteoporotic fracture** |  |  |  |  |  |  |
| Smoker (previous or current) | - | - | - | 26172 | 1.01 (0.94,1.09) | 0.78 |
| Non-smoker | - | - | - | 25723 | 0.93 (0.86,1.01) | 0.10 |
|  |  |  |  |  |  |  |
| **Hip arthroplasty** |  |  |  |  |  |  |
| Smoker (previous or current) | 24054 | 0.95 (0.90,1.00) | 0.07 | - | - | - |
| Non-smoker | 35446 | 0.99 (0.95,1.05) | 0.91 | - | - | - |
|  |  |  |  |  |  |  |
| **Knee arthroplasty** |  |  |  |  |  |  |
| Smoker (previous or current) | - | - | - | 26172 | 0.95 (0.89,1.01) | 0.08 |
| Non-smoker | - | - | - | 25723 | 0.90 (0.83,0.96) | 0.003 |
|  |  |  |  |  |  |  |
| Alcohol consumption |  |  |  | - | - | - |
| Daily | 9777 | 0.94 (0.84,1.06) | 0.31 | - | - | - |
| 3-4 times per week | 12163 | 0.93 (0.84,1.03) | 0.16 | - | - | - |
| <4 times per week | 37560 | 0.91 (0.86,0.95) | <0.001 | - | - | - |
|  |  |  |  |  |  |  |
| Physical activity | - | - | - |  |  |  |
| < median (2 hours/week) | - | - | - | 32253 | 0.93 (0.88,0.99) | 0.02 |
| ≥ median (2 hours/week) | - | - | - | 19642 | 0.92 (0.86,0.99) | 0.02 |
|  |  |  |  |  |  |  |
| Ethnicity |  |  |  |  |  |  |
| White | 55282 | 0.91 (0.87,0.95) | <0.001 | - | - | - |
| BAME | 4218 | 1.00 (0.84,1.20) | 0.97 | - | - | - |
|  |  |  |  |  |  |  |

**Supplementary Figure 1a:** Distribution of raw LTL

**Supplementary Figure 1b:** Distribution of z standardised LTL

**Supplementary Figure 2:** Scatterplot of relationship between LTL and chronological age


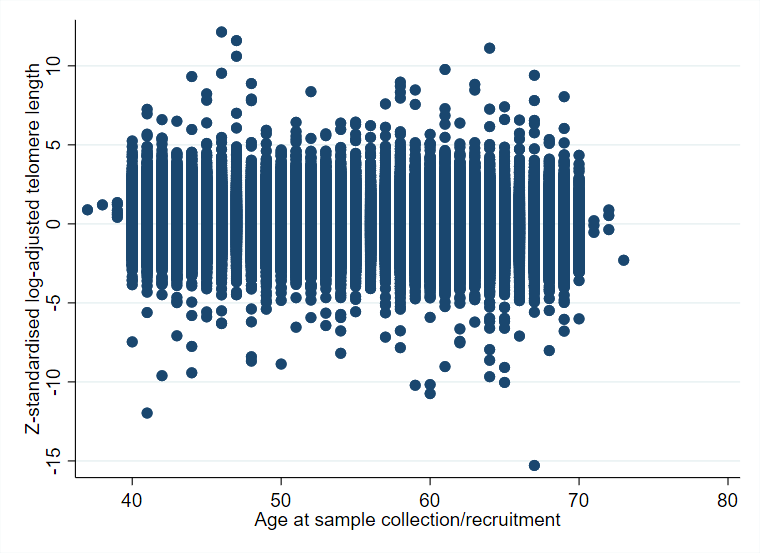

Supplement: Supplementary file 1 — Appendix S1 Supplementary Information Fig. S1 Fig. S2 Table S1 Table S2 Table S3 Table S4 Table S5 Table S6 Table S7 Table S8 Table S9 [file JBMR-37-1997-s001.docx]
